# Supplementary material for: Arabidopsis ASYMMETRIC LEAVES2 and Nucleolar Factors Are Coordinately Involved in the Perinucleolar Patterning of AS2 Bodies and Leaf Development
Source: Plants (Basel). 2023 Oct 19;12(20):3621. doi: 10.3390/plants12203621 (PMC10610122; doi:10.3390/plants12203621)
Supplement: Supplementary file 1 [file plants-12-03621-s001.zip › plants-2625913-supplementary.pdf]

Figure S1

A

Col-0 | *snp1* | *as2-1* | *as2-1* x *snp1* F_1

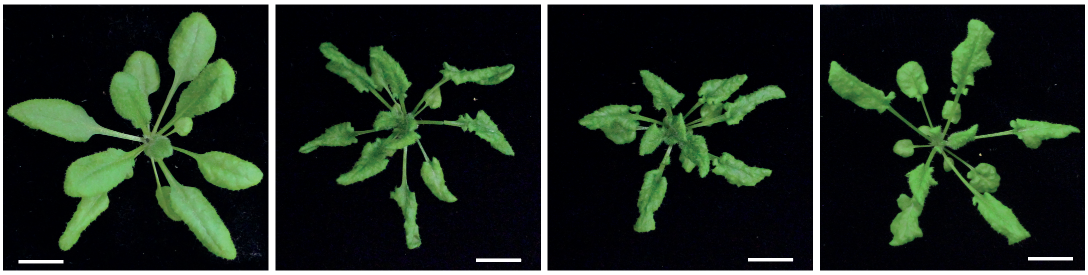

B

Fifth leaf	Leaf lobe	Leaflet-like structure
Col-0	0/26 (0%)	0/26 (0%)
as2-1	21/24 (87.5%)	3/24 (12.5%)
as2-1 x *snp1* F_1	19/24 (79.2%)	1/24 (4.2%)
snp1	21/29 (72.4%)	0/29 (0%)

Sixth leaf	Leaf lobe	Leaflet-like structure
Col-0	0/26 (0%)	0/26 (0%)
as2-1	22/24 (91.7%)	4/24 (16.7%)
as2-1 x *snp1* F_1	24/24 (100%)	2/24 (8.3%)
snp1	24/29 (100%)	0/29 (0%)

C

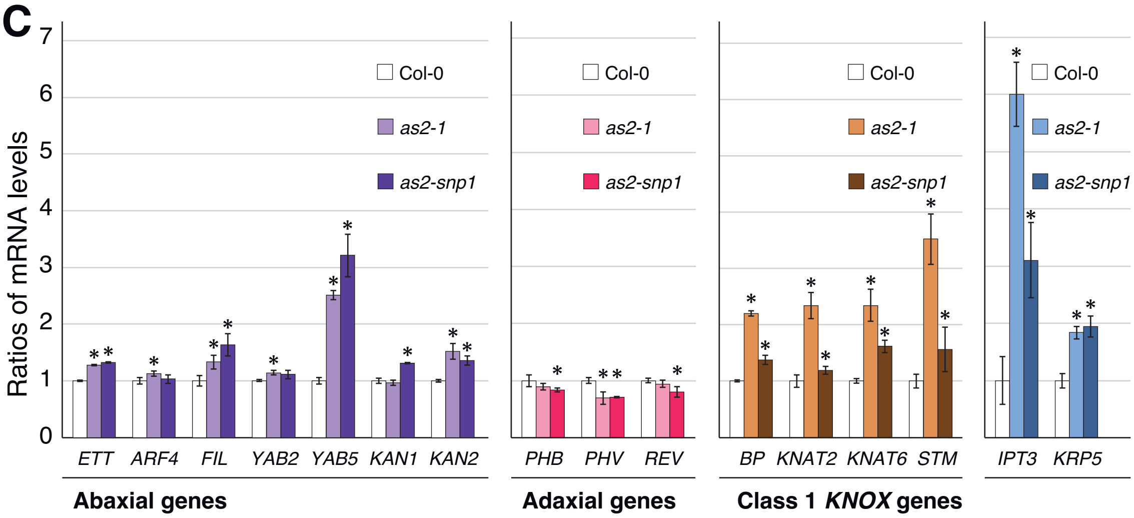

D

Ler (WT) | *as2-5*

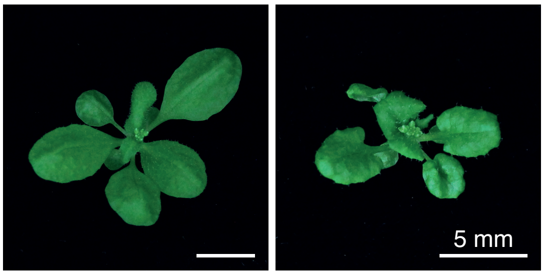

Figure S1. ***as2*** **mutants with base substitutions resulting in amino acid replacements in AS2/LOB domain.** (**A**) Leaf phenotype of *snp1* plants at 35 DAS. Bars, 5 mm. (**B**) The shapes of the fifth and sixth leaves of *snp1* plants at 35 DAS. (**C**) Levels of transcripts in the shoot apices of Col-0, *as2-1,* and *as2-snp1* mutants. Plants were grown at 22°C. Total RNA was prepared from shoot apices of 14-day-old plants, and transcript levels were examined by quantitative real-time RT-PCR. Each value was normalized by reference to the level of ACTIN (*ACT2*, At3g18780) transcripts. The values from Col-0 plants were set arbitrarily at 1.0. Bars indicate the s.d. from more than three biological replicates. White-purple-colored graphs: abaxial genes; white-pink-colored graphs: adaxial genes; white-brown-colored graphs: class 1 *KNOX* genes, white-blue-colored graphs: *IPT3* and *KRP5*. Significant differences from wild type were evaluated by Student's t-test and are represented by asterisks (*P<0.01). (**D**) Leaf phenotype of *as2-5* plants at 25 DAS.

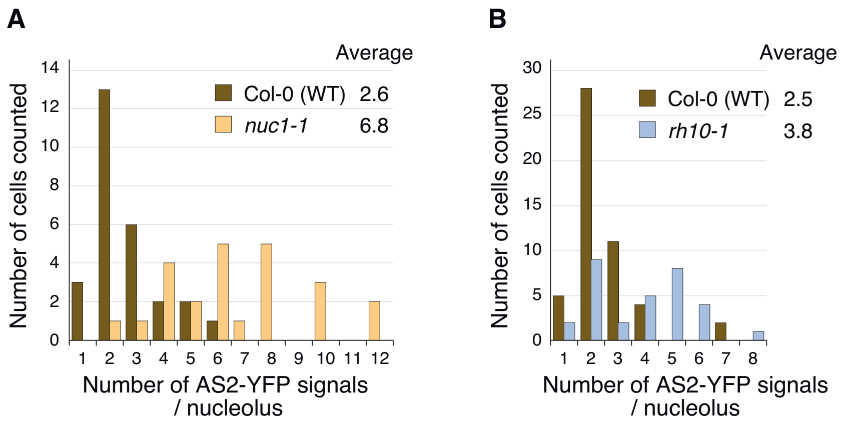

Figure S2. Distribution patterns of the number of AS2-YFP signals in nucleolus of the *nuc1-1* and *rh10-1* mutants. Expression of AS2-YFP was induced by incubating 6-day-old transgenic Arabidopsis plants (6 days after sowing) with 0.05 µM of 17β-estradiol for 16 h at 22°C. (**A**) Signals due to YFP in nucleolus were counted in Col-0 (27 cells) and *nuc1-1* (24 cells). (**B**) Signals due to YFP in nucleolus were counted in Col-0 (50 cells) and *rh10-1* (31 cells).

XhoI-AS2(CmASL3(Q21T_P22S_E23D))-3xGly-NcoI

GGGGCTCGAGATGGCATCTTCTTCAACAAACTCACCATGCGCCGCTTGCAAATTCCTCCG
GCGAAAATGTACGTCAGACTGTGTATTCGCGCCCTATTTCCCACCGGACCAGCCACAAA
AATTCGCAAACGTTCACAAAGTGTTTGGAGCAAGTAACGTGACAAAGCTCCTCAACGAG
CTTCACCCTTCACAACGTGAAGACGCAGTGAACTCTTTGGCCTATGAAGCCGACATGCG
CCTCCGTGACCCTGTCTACGGCTGCGTCGGCGTCATCTCTCTCCTCCAACATCAGCTTCGT
CAGCTTCAGATAGATCTCAGCTGTGCTAAATCTGAGCTCTCTAAGTACCAAAGCCTCGGT
ATCCTCGCCGCCACTCATCAGAGTCTTGGCATCAACTTACTCGCCGGAGCAGCAGATGGA
ACAGCCACCGCCGTGAGAGACCACTATCACCACCACCAGTTTTTCCTAGAGAACAAAT
GTTTGGTGGCTTGGATGTTCCGGCCGGTAACAACTACGACGGTGGGATTCTTGCCATTGG
ACAGATCACTCAGTTTCAGCAGCCGAGAGCCGCCGCTGGAGATGATGGTCGCCGTACTG
TTGATCCGTCTGGAGGTGGCGCCATGGGGAA

XhoI-AS2(E23D)-3xGly-NcoI

GGGGCTCGAGATGGCATCTTCTTCAACAAACTCACCATGCGCCGCTTGCAAATTCCTCCG
GCGAAAATGTCAACCGGACTGTGTATTCGCGCCCTATTTCCCACCGGACCAGCCACAAA
AATTCGCAAACGTTCACAAAGTGTTTGGAGCAAGTAACGTGACAAAGCTCCTCAACGAG
CTTCACCCTTCACAACGTGAAGACGCAGTGAACTCTTTGGCCTATGAAGCCGACATGCG
CCTCCGTGACCCTGTCTACGGCTGCGTCGGCGTCATCTCTCTCCTCCAACATCAGCTTCGT
CAGCTTCAGATAGATCTCAGCTGTGCTAAATCTGAGCTCTCTAAGTACCAAAGCCTCGGT
ATCCTCGCCGCCACTCATCAGAGTCTTGGCATCAACTTACTCGCCGGAGCAGCAGATGGA
ACAGCCACCGCCGTGAGAGACCACTATCACCACCACCAGTTTTTCCTAGAGAACAAAT
GTTTGGTGGCTTGGATGTTCCGGCCGGTAACAACTACGACGGTGGGATTCTTGCCATTGG
ACAGATCACTCAGTTTCAGCAGCCGAGAGCCGCCGCTGGAGATGATGGTCGCCGTACTG
TTGATCCGTCTGGAGGTGGCGCCATGGGGAA

Figure S3. The DNA sequences synthesized. as2(Q21T_P22S_E23D) and as2(E23D) DNA were synthesized and cloned in the EcoRV site of pUC by GenScript (Tokyo, Japan). The DNA sequences synthesized were described below. *Xho* I site was added upstream of the *AS2* start codon, and the codons that code for three Glycine and one Alanine as a linker and *Nco* I site were added downstream of *AS2*. The ATG colored with red indicates the start codon of AS2. The DNA sequences colored with yellow encode the three amino acid residues, which were replaced in as2(Q21T_P22S_E23D) and as2(E23D) variants, and the sequences colored with blue-green encode the four cysteine residues in the ZF-motif. The *Xho* I site is colored with cyan and the *Nco* I site is colored with purple.

Table S1 Sequences of oligomers used for real-time RT-PCR, construction, genotyping, AlphaScreen and pull-down assays

1 The primers used for real-time PCR (Takahashi et al., 2013; Matsumura et al., 2016)

name	sequence
ACTIN2-F	5'- TCGGTGGTTCCATTCTTGCT -3'
ACTIN2-R	5'- GCTTTTTAAGCCTTTGATCTTGAGAG -3'
ETT-F	5'- ATCATTGAGATTCCAGAGGGTCTT -3'
ETT-R	5'- GGCTCCACCATCCGAACA -3'
ARF4-F	5'- CAGGTGTTATGGACCTGGATAGG -3'
ARF4-R	5'- CCAGCAAATTGCGGGAAT -3'
KAN1-F	5'- CCACGCGCGGTTTGTT -3'
KAN1-R	5'- GACTTTGGSGTTGCTCTTTCA -3'
KAN2-F	5'- AAGGAACTAGATGGAAAGTGCTCAA -3'
KAN2-R	5'- GCTTGTTCCCGAGATGCTTG -3'
FIL-F	5'- GCCCACTTCCCCCACATAC -3'
FIL-R	5'- TTGGTTTTCTTCACGGGTTGA -3'
YAB5-F	5'- ACGCCCTAATTTCCAGGCAAC -3'
YAB5-R	5'- GTTGCTCAGTTATGGTACGAG -3'
PHB-F	5'- GCTGTTGACTGGGTTCAGATGA -3'
PHB-R	5'- GCGAAATAGCGACTATGCCAAT -3'
PHV-F	5'- GTGAAACAGCTACGATACCAATAGAATC -3'
PHV-R	5'- CCTTTGCAAGGCTACAGGAACT -3'
REV-F	5'- ATCCAAAGTCGTTGCACAAAAA -3'
REV-R	5'- GACTCTTGGGCTAATTGCCTGAT -3'
BP-F	5'- TGTTGTTTCCACATATGAGCTCTCT -3'
BP-R	5'- TCATGATCAGATCGGAAGCAAT -3'
KNAT2-F	5'- TTCCGCTCGACGGAAGAC -3'
KNAT2-R	5'- AATCGGACGGCATCATCAAC -3'
KNAT6-F	5'- GATGTCACCGGAGAGTCTCATG -3'
KNAT6-R	5'- CGGCGGAGGAACATAGCA -3'
STM-F	5'- CTCCTCCCCAAGGAACTAAGAAC -3'
STM-R	5'- TCCTCCTGCAACGATTTCG -3'
IPT3-1F	5'- CCGCCTGAAGCCGACTTAA -3'
IPT3-1R	5'- TTTAGGACGGATTCAATGGAGAGA -3'
KRP2-F	5'- CGTGGATTTACGATGATTTGAA -3'
KRP2-R	5'- GCGGCGAGACTCTACATCTT -3'
KRP5-F	5'- TCCTAGTGTCAATCAATGTCAAACG -3'
KRP5-R	5'- CGTCGTATCCGGCTCTAATTTC -3'

2 The primers used for as2-variant-YFP construction

name	sequence
AS2(XhoI_1-)	5'- GGGGCTCGAGATGGCATCTTCTTCAACAAACTC -3'
AS2(-597_GGG_NcoI)	5'- TTCCCCATGGCGCCACCTCCAGACGGATCAACAGTACGG -3'
AS2(XhoI_22-)	5'- GGGGCTCGAGATGTCACCATGCGCCGCTTG -3'
AS2(XhoI_73-)	5'- GGGGCTCGAGATGGTATTCGCGCCCTATTTC -3'
AS2(-327_GGG_NcoI)	5'- TTCCCCATGGCGCCACCTCCGAGCTCAGATTTAGCACAGC -3'
T3	5'- AATTAACCCTCACTAAAGGG -3'
T7	5'- TAATACGACTCACTATAGGG -3'
pER8-ProF	5'- GTAATATGCTCGACTCTAGG -3'
pER8-TerR	5'- TGGTGTGTGGGCAATGAAAC -3'

3 The primers used for genotyping

name	sequence	
GFP F2	5'- CAGCTCGCCGACCACTAC -3'	for *EYFP*
GFP R2	5'- CTTGTACAGCTCGTCCATGCC -3'	
Nuc1-1GT_F1	5'- CTCAGTTCTCCATGGGAAAGTC -3'	for *NUC1*
Nuc1-1GT_R1	5'- AGAGACCTTACACGTTCATGGG -3'	
Nuc1-1GT_R2	5'- TATCATCTTCAGGCTCTCTCTTGCC -3'	for *nuc1-1*
RB06	5'- TTCCCTTAATTCTCCGCTCATGATC -3'	
YM-18	5'- ATATCGAGCCCTCTACTAGCCA -3'	for *RH10* and *rh10-1*
YM-19	5'- TCCTGTGAAGGTATGGAGACAC -3'	
dCAPS-rid2-F	5'- CCCTTAAATTTATGTGTGGC -3'	for *RID2* and *rid2-1*
rid2-Rv	5'- GCTCCGTCAATAACTCCAGAT -3'	

4 The DNA sequences of oligomers used for AlphaScreen & pull-down assays

name	sequence
biotin-Ex1_264	5'- CTCAGGGACATTTGGAACAAGCCCCCGATTTCTCCGCCGCGATTTACGGG -3'
nonlabel-Ex1_264	5'- CTCAGGGACATTTGGAACAAGCCCCCGATTTCTCCGCCGCGATTTACGGG -3'
antisense-Ex1_264	5'- CCCGTAAATCGCGGCGGAGAAATCGGGGGCTTGTTCCAAATGTCCCTGAG -3'
biotin-Ex1_264m	5'- CTCAGGGACATTTGGAACAAGCCCCCGATTTCTCATAATAGATTTACGGG -3'
nonlabel-Ex1_264m	5'- CTCAGGGACATTTGGAACAAGCCCCCGATTTCTCATAATAGATTTACGGG -3'
antisense-Ex1_264m	5'- CCCGTAAATCTATTATGAGAAATCGGGGGCTTGTTCCAAATGTCCCTGAG -3'
